# Distraction towards contextual alcohol cues and craving are associated with levels of alcohol use among youth

**DOI:** 10.1186/s12888-018-1919-0

**Published:** 2018-10-30

**Authors:** Timo Lehmann Kvamme, Kristine Rømer Thomsen, Mette Buhl Callesen, Nuria Doñamayor, Mads Jensen, Mads Uffe Pedersen, Valerie Voon

**Affiliations:** 1 Centre for Alcohol and Drug Research, School of Business and Social Sciences, University of Aarhus, Bartholins Allé 10, Building 1322, 2. Floor, Aarhus C, Denmark; 2 Department of Psychiatry, University of Cambridge, Cambridge, UK; 3 Center of Functionally Integrative Neuroscience, MINDLab, Aarhus University, Aarhus C, Denmark; 4 Department of Psychiatry and Psychotherapy, Charité – Universitätsmedizin Berlin, Berlin, Germany; 5 Behavioural and Clinical Neurosciences Institute, University of Cambridge, Cambridge, UK; 6 NIHR Biomedical Research Council, University of Cambridge, Cambridge, UK

**Keywords:** Alcohol, Distraction, Go/NoGo task, Craving, Zero-inflated negative binomial

## Abstract

**Background:**

Controlling drinking behaviour requires the ability to block out distracting alcohol cues in situations in which drinking is inappropriate or harmful. However, at present few studies have investigated whether distraction and response inhibition to contextual alcohol cues are related to alcohol use in adolescents and young adults. We aimed to investigate whether tendencies towards distraction and failures of response inhibition in the presence of contextual alcohol cues, and alcohol craving were associated with higher levels of alcohol consumption, beyond what could be explained by demographic variables.

**Methods:**

To test this, 108 participants (Mean age = 21.7, range = 16–27), whom were both drinkers and non-drinkers performed a modified Go/NoGo task tailored to measure distraction and response inhibition in the presence of alcohol cues relative to neutral stimuli. Alcohol craving was assessed using a visual analogue scale of craving for different types of alcohol cues. Levels of alcohol use and problematic alcohol use were assessed using a self-report measure of number of drinking days in the previous month and the Alcohol Use Disorders Identification Test. Data were analysed using sequential multiple regression using a zero-inflated negative binomial distribution model.

**Results:**

Drinking days correlated with distraction but not response inhibition to contextual alcohol cues. Sequential regression analyses revealed that the inclusion of distraction bias accounted for 11% additional variance (significant) in alcohol use, in addition to that explained by demographics alone (17%). Craving for alcohol explained an additional 30% variance (significant) in alcohol use.

**Conclusions:**

The results reported here support the idea that both biased distraction towards alcohol cues and alcohol craving are associated with preceding drinking days, but not necessarily drinking status. Further studies are warranted that address whether cognitive distraction to alcohol-related cues cause or is an effect of alcohol use among youth.

## Background

Goal-directed behaviour can become derailed by momentary distracting stimuli such as the sight of alcohol cues [1]. Importantly, these alcohol cues are often conceptualised as acquiring their incentive value long before an individual is diagnosed with any Alcohol Use Disorder (AUD; American Psychiatric Association, 2013) [2, 3]. Initial studies on cognitive biases reported an association between attentional and approach biases to alcohol cues and indices of (problematic) alcohol consumption [4–6]. These cognitive biases have been hypothesized to be central to the aetiology and maintenance of addictive behaviours [7, 8].

Attentional biases towards alcohol cues have traditionally been measured using the Stroop task, in which participants are to report the colour of a word while ignoring its meaning (which is either substance-related or neutral). Here, heavy drinkers tend to be slower than light drinkers in reporting the colours of alcohol-related words compared to neutral words [1, 9, 10], suggesting that heavy drinkers are distracted by the presence of alcohol-related cues. In the visual-dot probe task heavy drinkers show an attentional bias indicated by reduced latencies when detecting a target that replaces an alcohol-related cue [11]. Furthermore, studies suggest that heavy drinkers display attentional biases in later stages of processing whereas abstinent alcoholics differ in initial orientation [12, 13]. Despite these findings, the purported ability of the attention bias measure to predict alcohol relapse [6] has failed replication [14, 15] and a recent review argue that the clinical relevance of attentional biases may be overstated in the literature [16].

Responses towards alcohol cues have also been investigated in the alcohol-shifting task, a variant of the Go/NoGo paradigm. In an alcohol-shifting task, the participant is asked to switch between performing two separate tasks: 1) to associate alcohol cues as Go-signals vs. neutral stimuli as NoGo-signals and 2) neutral stimuli as Go-signals and alcohol cues as NoGo-signals. Here, Noël et al. reported that both alcohol dependent and control participants were faster to respond to alcohol-related words when they were Go-signals as opposed to neutral words [17]. It was reported that alcohol dependent individuals made a higher number of false alarms (Go responses to a NoGo signal) and omission errors (NoGo responses to a Go signal) when alcohol stimuli had to be detected compared with control participants. Mixed results emerge when problem drinkers and individuals who display moderate to heavy alcohol use (but without a diagnosis of alcohol dependence) are tested. Rose and Duka report in a single sample that moderate-to-heavy drinkers have slowed responses to alcohol Go-signals [18]. In contrast, Adams et al. show faster responses to alcohol Go-signals, with no clear difference between heavy and light drinkers defined by quantity of consumption [19]. Faster responses and a higher rate of false alarm to alcohol Go-signals in general were also reported in a population of problem drinkers defined by problematic drinking severity scores [20].

The alcohol and neutral stimuli in the aforementioned variant of the Go/NoGo task can be considered task-relevant as participants are instructed to form the association between the detection of either alcohol or neutral stimuli and a motor response or inhibition. A demonstration of faster responses and a higher rate of false alarm to alcohol go-signals [20], could potentially arise from multiple cognitive processes. The alcohol stimuli could have acquired attention-grabbing properties akin to a cue-specific attention bias speeding up the detection of the target. Alternatively, the alcohol stimuli could induce enhanced motivational processes due to the associated positive affect leading to hastened motor responses as seen with approach biases [5]. In both cases the resultant overt behaviour is the same; hastened responses and a higher rate of false alarm in the presence of alcohol.

Less explored is the option to use neutral and alcohol stimuli as task-irrelevant distractors, similar to an Emotional Stroop task [1]. In this version of the Go/NoGo task which is also modified with alcohol cues, instructions remains the same as participants are to detect a target (either the letter “P” or “F”) as Go or NoGo targets, while task-irrelevant alcohol or neutral stimuli interferes with the detection of the targets [21]. Here, the task models an everyday scenario, where one is searching for a signal relevant for goal-directed behaviour while contextual salient cues interact with the cognitive process. To our knowledge, an assessment of alcohol-specific cognitive bias using this task with pictorial stimuli has not been reported in the scientific literature.

This approach is theoretically intriguing as generalized Pavlovian-instrumental-transfer theories would argue that the presence of the motivational characteristics of contextual alcohol cues might enhance the process of detection, thus hastening responses in the presence of alcohol stimuli [22, 23]. Another set of theories would argue that emotional distractors can interfere with on-going cognitive processes [24, 25]. As an example, a recent study employing distractors in a Go/NoGo task suggests that emotional cues exert distracting effects with task-irrelevant cues [26]. We propose that the repeated pairing of alcohol cues and alcohol consumption, as a rewarding outcome, might result in alcohol cues gaining emotional valence and attention-grabbing properties thus slowing responses in the presence of alcohol stimuli [27, 28].

According to the incentive-salience model, the relationship between consumption and biases towards drug-cues is not limited to the above-mentioned cognitive bias measures, but also include evaluative biases such as self-reported craving of the drug of abuse (a subjective proxy of the motivational state) [7, 8]. Studies on alcohol use are plagued by contrasting findings as some studies show higher craving scores in heavy compared to light drinkers [13] as well as a correlation between craving for alcohol and drinks/week in individuals suffering from AUDs [29]. Other studies have, in contrast, not found correlations between craving and alcohol use in social drinkers [3] and heavy drinkers [30].

As increasing levels of alcohol consumption during adolescence and young adulthood may lead to the later emergence of AUDs [2, 31], an investigation of how these cognitive and evaluative biases are associated with heightened alcohol consumption in this age-group is important.

In this study, we devised a modified Go/NoGo task in order to investigate attentional distraction and response inhibition to contextual alcohol cues and the relationship to past month drinking days in a sample of adolescents and young adults. The first aim of the study was to test for a main effect of alcohol-related distractors on response latencies and false alarms to no-go signals. On the basis of other findings [1, 26], we expected alcohol cues to act as distractors due to their elevated emotional/incentive value compared to neutral cues leading to slower reaction times and a higher percentage of false alarms. We operationalised an alcohol-specific distraction and inhibition bias as slowed responding and increased false alarms on trials with task-irrelevant alcohol cues compared with task-irrelevant neutral stimuli. Secondly, the study aimed to investigate the association between cognitive biases in the modified Go/NoGo Task and drinking days in the preceding month. We expected the retrospective alcohol consumption in the weeks leading up to cognitive testing to correlate with indices of biased distraction and inhibition towards alcohol cues. Moreover, we expected cognitive biases to explain a larger degree of variance in levels of alcohol use than what can be attributed to demographic variables of age, gender, and years of education. Finally, we explored whether craving for alcohol could explain any additional variance in levels of alcohol use.

## Methods

### Participants

The present study is part of a larger study designed to examine risk factors for problematic use of substances and non-substances among youth (for details see Rømer Thomsen et al., 2018) [36]. In order to obtain a subclinical group of youth with varying risk of developing problematic use, and with varying levels of current use, we recruited adolescents and young adults with varying degree of externalizing behavior problems. Externalizing behavioural problems have been consistently shown to increase the risk of problematic substance use and have been associated longitudinally with problematic use of alcohol and other substances [32–34].

Participants were selected from a representative national survey (*YouthMap2014 by Statistics Denmark*, *N* = 3064) based on their level of externalizing problems, measured with the EP6 [32] (EP6 consists of six items that identify externalizing behaviour problems). In total, we included 109 adolescents and young adults (aged 15–26 years) with varying levels of EP6: no externalizing problems (*N* = 34), minimal externalizing problems (*N* = 19), moderate externalizing problems (*N* = 25), and severe externalizing problems (*N* = 30).

Participants were included if they had no current major psychiatric disorder assessed with the Mini International Neuropsychiatric Inventory [35], and did not receive medication. Participants were instructed to abstain from substances (tobacco was allowed) at least 24 h prior to their participation. The study was approved by the local ethics committee and all participants received verbal and written information about the study and gave written consent prior to inclusion. Furthermore, in accordance with requirements by the local ethics committee (De Videnskabsetiske Komitéer for Region Midtjylland, case number: 1–10–72-123-15), for participants aged 15–17, parents also received written information about the study (and were offered verbal information) to ensure that the adolescent’s consent was given under parental supervision.

### Self-report measures

#### Drinking days last 30 days

We measured alcohol use by asking participants to report the number of days in which they had drunk alcohol the past 30 days as part of the Addiction Severity Index [37] also used in the European Model Questionnaire, EMCDDA [38]. We regard reporting the number of days of drinking in the recent month to be less susceptible to errors of memory and other self-report response biases [39].

AUDIT scores. Problematic use of alcohol was measured using the Alcohol Use Disorder Identification Test (AUDIT) [40], which is a 10-item questionnaire developed as a screening instrument for hazardous and harmful alcohol consumption.

#### Craving scores & personalization

Participants were presented with 40 alcohol pictures and instructed to rate how much they were tempted to drink this right now (In Danish; “hvor fristet er du til at drikke dette”) on a computerized Visual Analog Scale from 0 to 9. It was our intention that participants would be exposed to the alcohol cues and asked to reflect on their level temptation to drink, in order to prime any attentional-distraction bias they might have to the cues.

The alcohol pictures were obtained from the internet and aimed to reflect the array of alcohol choices that are typically available to Danish youths. The alcohol pictures included bottles of beer, cider, alcopops and wine, and glasses of beer, wine, liquor and cocktails. The purpose of the picture rating was to derive a composite craving score and to select a subset of the 10 highest rated images that could be used in the subsequent Go/NoGo task.

### Go/NoGo task

**Fig. 1:**
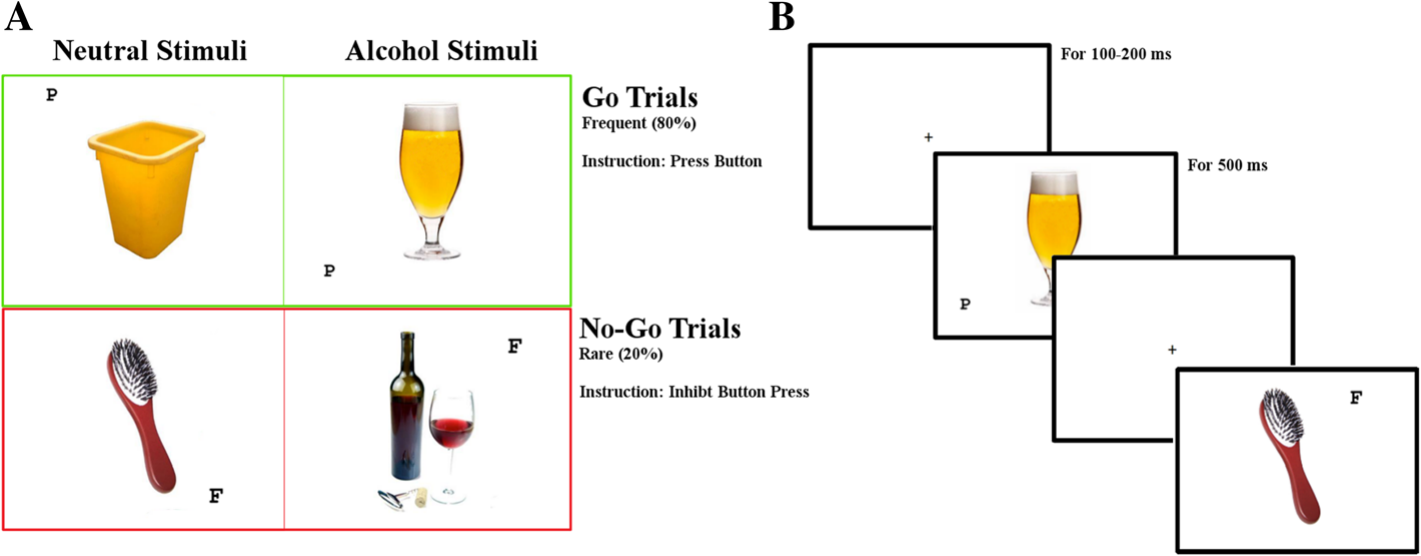
Alcohol Modified Go/NoGo Task. **a** Table representation of the different trial types, with either equally occuring neutral or alcohol stimulus type presentations combined with either frequently occuring Go or rare NoGo trials. **b** Example of trial sequence of two trials interspersed with 100–200 miliseconds (ms) intertrial intervals

Distraction and response inhibition bias to alcohol cues was measured using a modified version of the Go/NoGo Task, similar to that of Houben et al. [21]. Participants were instructed to press the space bar when Go-signals were presented and withhold responding when NoGo-signals were presented. The Go/NoGo-signals were the letters “P” and “F” (counterbalanced across participants), displayed randomly but with equal occurrence in one of the four corners of a centred image. A task-irrelevant distractor of either a neutral or an alcohol cue were presented in the centre of the screen (Fig. 1). The alcohol cues were individualized based on participants 10 highest-rated pictures. Each alcohol picture had been matched to a neutral picture obtained from a set of standardized pictures [41], approximately matched for colour, form, and complexity. Images subtended a visual angle of 8.5° × 13.7°, and letters were presented at 6.7° of retinal eccentricity subtending 0.7° × 0.7°. Stimuli were presented against a white background on a 19″ monitor (1280 × 1024 resolution, 60 Hz).

Participants were seated in a dimly-lit room and wore headphones for sound-attenuation. All participants completed 10 practice trials without alcohol cues. In the main task, each of the 10 different alcohol and neutral images was paired 16 times with a Go-signal (80% of trials) and 4 times with a NoGo-signal (20% of trials); totalling 400 trials taking approximately 8 min. Trials were presented in a random order.

Stimulus duration was 500 ms, the inter-trial interval was a random interval between 100 and 200 ms where a central fixation cross was displayed. If participants missed the 500 ms reaction time (RT) deadline, a 300 ms error tone of 440 Hz was delivered through the headphones accompanied by a visual feedback text “you were too slow” presented for 500 ms. False alarms (commission errors) did not lead to any feedback except in the initial practice trials where a “wrong” text was presented. The stimulation interface was custom programmed in Python using PsychoPy (Version 1.81.0) [42].

### Analysis strategy

Data analyses were performed using the statistical programming language R (Version 3.4.3). Out of the 109 participants, one participant did not complete the task. In line with prior research on cognitive biases using RT-based measures, we calculated medians to summarise participants’ first level scores on the Go/NoGo task as medians minimize the effect of outliers [43, 44].

Distraction bias scores were calculated by subtracting the median RT of alcohol Go trials from neutral Go trials. To control for difference in average RT, we used the improved scoring-algorithm by Greenwald and colleagues (2003) which standardizes the bias scores by dividing the individual RT difference by a personalized standard deviation of these latencies [45]. False alarm rates were calculated for alcohol and neutral trials using signal detection theory [46]. Response inhibition bias was computed by subtracting the false alarm rate on neutral NoGo trials from the false alarm rate on alcohol cued NoGo trials. Positive bias scores indicate a tendency towards distraction or inhibition failures in the presence of alcohol cues.

Craving scores were derived from the median of the same 10 highest rated alcohol images as used in the task. We reasoned that the 10 highest rated images would likely reflect their choice of beverage if they consumed alcohol.

On the second level, the aforementioned variables were examined for outlier participants using the R package ‘mvoutlier’ [47], which allows for the robust evaluation of multivariate datasets. We also inspected the values that were 2.5 standard deviations (SD) from the group mean. Both approaches resulted in the detection and removal of two outliers, i.e. two participants who reported 22 and 27 days drinking in the last 30 days, reducing the final sample to 106.

### Statistical tests

One-sample t-tests were used to evaluate whether bias scores significantly deviated from zero. Pearson’s correlations and sequential multiple regression analyses were used to determine the association between demographics, behavioural task measures, and alcohol use.

Despite the relative prevalence of alcohol use, many adolescents and young adults have not consumed alcohol in the last 30 days, with the underlying cause often being unknown. Consequently, a challenge in modelling alcohol consumption is to appropriately account for the large number of zeroes (potentially abstinent individuals), along with a long right tail of heavier drinkers [48]. In our analysis, the days of alcohol use within the last 30 days were not normally distributed (Shapiro-Wilk test: *p* < 0.001) and consisted of a large number of zeros (18.7%). Thus, we employed a generalized linear model (GLM). GLMs extend the ordinary least squares (OLS) regression to instances where the distribution of the response variable is non-normal and allows the magnitude of the variance to be modelled through the use of appropriate ‘link’ functions. Similar to other modelling approaches [49], the distribution model for alcohol use was selected after inspection of relevant histograms and QQplots, and comparison of fitted models using the Akaike information criterion (AIC).

Accordingly, the Zero-Inflated Negative Binomial (ZINB) distribution model was chosen with a logit link function consisting of a natural offset boundary at 30. The primary strength of the ZINB regression model is the explicit partitioning of the zero values into two types: “structural” zeros, i.e. those that occur because a participant has chosen to be abstinent and those zeros that occur from mere chance among eligible drinkers. The model thus simultaneously estimates a logistic component of the odds ratio of a participant being classified as a structural zero and a count component reflecting the levels of consumption along a scale from 0 to 30 days.

To assess whether distraction bias scores explained variance of alcohol use beyond what could be attributed to demographic variables, sequential multiple regression using ZINB distribution models was employed. To control for other variables, in the first step the dependent variable of alcohol use measured as drinking days within the last 30 days was regressed onto demographic variables of age, gender and years of education (Model 1), after which distraction bias was entered in a second step (Model 2). In a third step (Model 3) we explored whether the addition of craving scores significantly explained further variance in drinking days. We did not include interactions. We performed model comparisons of the difference in the log-likelihood ratio between sequential ZINB models that did or did not include additional variables of interest. This ratio approximates a χ^2^ distribution and can thus be compared using a likelihood ratio chi-squared test (LR χ^2^) to assess whether two models differ significantly, where the degrees of freedom are the difference in the free parameters of the two models. Associated *p*-values of the regression models were Bonferroni-corrected for multiple comparisons and the results were considered significant if *p* < 0.05. Regression diagnostics indicated no violation of the assumptions of multicollinearity, independence and normal distribution of errors (maximum Cook’s distance = 0.036, maximum deviance residual = 2.23). Variance inflation factors were only above 1.20 for age and years of education which were control variables, thus suggesting that multicollinearity was not a problem. The model did violate the assumption of homoscedasticity (Breusch-Pagan test: *p* = 0.018), although this is common for count models [48, 50].

To obtain effect sizes for explained variance in the regression models we compared the likelihood ratio-based *R*^2^, which is a measure of fit analogous to the coefficient of determination (*R*^2^) in OLS regression [51]. Although the likelihood ratio-based *R*^2^ approximates an easily interpretable measure of effect size, the *R*^2^ used in maximum-likelihood estimation regression is different from the *R*^2^ used in OLS regression and should be interpreted with caution [52]. When estimating the total variation explained by a model, we compare the *R*^2^ to a null model, which only models the dependent variable with an intercept.

## Results

**Fig. 2:**
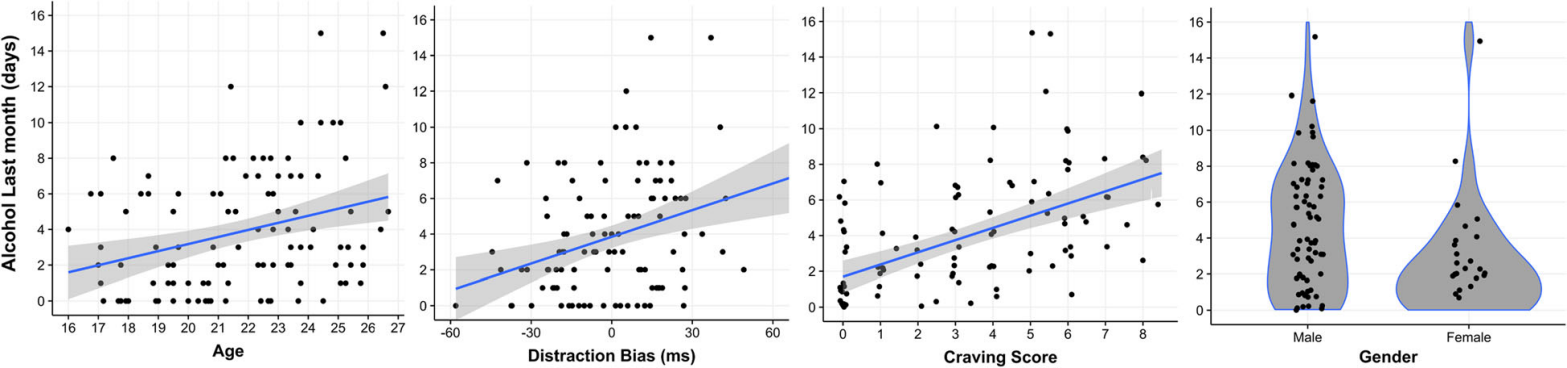
Scatter plots and Violin plots of variables associated with drinking days. Each dot in each plot represents the data from one participant. The blue lines in the scatter plots show the fit of a linear model, and the gray area indicates the standard error. Dots on the plots of craving scores and gender have been jittered (0.1) to avoid overlapping dots. A scatter plot of years of education is not included due to resemblance with the age plot. ms = milliseconds

We found no main stimulus differences in either distraction (*t*_105_ = 0.10, *p* = 0.91) or response inhibition (*t*_105_ = 0.047, *p* = 0.96) as bias scores did not significantly deviate from zero. Pearson’s correlation coefficients are presented in Table 1 and visualized in scatter plots (Fig. 2). The distraction bias scores were moderately correlated with drinking days within the last 30 days (*r* = 0.33, *p* < 0.001), but did not correlate with AUDIT scores (*r* = − 0.10, *p* = 0.28). There were no statistically significant correlations between response inhibition bias and alcohol use the last 30 days or problematic use (AUDIT). Instead, craving scores were strongly correlated with drinking days (*r* = 0.51, *p* < 0.001) and moderately correlated with problematic use (AUDIT) (*r* = 0.36, *p* < 0.001) as well as distraction bias (*r* = 0.22, *p* = 0.02).

**Table 1:** Summary Statistics and Pearson’s correlation coefficients

	M [SD]	1	2	3	4	5	6	7	VIF
1. Alcohol Use (Drinking Days)	3.86 [3.44]	**1.00**							–
2. Distraction Bias	0.22 [22.41]	0.33***	**1.00**						1.06
3. Inhibition Bias	0.0004 [0.10]	0.00	0.04	**1.00**					1.03
4. AUDIT	8.65 [5.89]	0.23*	− 0.10	0.00	**1.00**				–
5. Craving	3.16 [2.57]	0.51***	0.22*	0.10	0.36***	**1.00**			1.12
6. Gender^1^	f/m 0.43%	−0.26**	− 0.02	0.07	− 0.12	−0.22*	**1.00**		1.14
7. Age	21.71 [2.69]	0.31**	−0.01	0.00	0.08	0.00	−0.08	**1.00**	1.85
8. Years of Education	13.50 [1.86**]**	0.20*	−0.06	0.07	0.08	0.01	0.09	0.66***	1.87

^a^Gender was coded as male = 0, female = 1; M = mean; SD = standard deviation; Numbers from 1 to 7 in the top row represents each variable in the leftmost column; *VIF* Variance Inflation Factors for the sequential regression models of drinking days, *AUDIT* Alcohol Use Disorder Identification Test; Significant coefficients are in **boldface.** *p < 0.05, **p < 0.01, *p* < 0.001***

Model 1 which was constituted by control variables explained 16.7% of the variance in drinking days which was significantly more than the null model (LR*χ*^2^(2) = 19.14, *p* = 0.003). The explained variance in Model 2, which included distraction bias, explained an additional 11.0% and was significantly higher than Model 1 (LR *χ*^2^(2) = 12.25, *p* = 0.002; see Table 2). Model 3, which included craving scores in addition to distraction bias and control variables, explained an additional 30.2% variance in drinking days, which was significantly different from Model 2 (LR *χ*^2^(2) = 37.32, p < 0.001).

**Table 2:** Zero Inflated Negative Binominal Models of Alcohol Use

	Logistic Component				Count Component			
	β [SE]	Exp(β)	Z	P	β [SE]	Exp(β)	z	p
**Model 1:** Change *R*^2^: 0.17 LR *χ*^2^(6) = 19.14^**^ (AIC: 514.62)								
Age	−0.248[0.204]	0.780	− 1.217	0.224	0.559[0.036]	1.057	1.540	0.124
Gender	0.652[0.792]	1.919	0.823	0.411	−0.469[0.192] *	**0.626**	**− 2.434**	**0.015**
Years of education	−0.037[0.280]	0.964	−0.133	0.894	0.021[0.050]	1.021	0.411	0.681
**Model 2:** Change *R*^2^: 0.11 LR *χ*^2^(2) = 12.25^**^ (AIC: 506.36)								
Age	−0.224[0.199]	0.799	−1.128	0.259	0.051[0.034]	1.052	1.510	0.131
Gender	0.685[0.796]	1.983	0.860	0.390	−0.460[0.182] *	**0.631**	**−2.534**	**0.011**
Years of education	−0.079 [0.280]	0.924	−0.281	0.779	0.031[0.047]	1.031	0.660	0.509
Distraction Bias	−0.024[0.016]	0.976	−1.474	0.141	0.010[0.003] **	**1.009**	**2.910**	**0.004**
**Model 3:** Change *R*^2^: 0.30 LR *χ*^2^(2) = 37.32^***^ (AIC: 473.05)								
Age	−0.878[0.412] *	**0.416**	**−2.130**	**0.032**	0.065[0.031] *	**1.067**	**2.086**	**0.037**
Gender	−1.668[1.564]	0.188	−1.066	0.286	−0.375[0.160] *	**0.687**	**−2.340**	**0.019**
Years of education	0.543[0.526]	1.721	1.032	0.302	0.033[0.043]	1.034	0.771	0.441
Distraction Bias	−0.054[0.038]	0.948	−1.426	0.154	0.109[0.028] **	**1.008**	**2.811**	**0.005**
Craving Scores	−4.099[2.365]	0.017	−1.733	0..083	0.109[0.029] ***	**1.115**	**3.802**	**< 0.001**

Note. *N* = 106, Gender was coded as male = 0, female = 1; Significant coefficients are in **boldface.** *p < 0.05, **p < 0.01, *p* < 0.001***. Final model *R*^2^: 0.58, adjusted *R*^2^: 0.48. The *R*^2^ is a log-likelihood-based coefficient of determination (see method for details). *LR χ*^*2*^*(df)* Log Likelihood Ratio Chi-squared test, *df* degrees of freedom, *β* coefficients *SE* standard error, *Exp(β)* exponentiated coefficient. Exponentiated coefficient represent the odds of a structural zero score in the logistic component of the model and levels of use in the count component of the models

Concerning the statistical significance of individual variables of the ZINB models, in Model 1, only gender was associated with drinking days, whereby expected male users tended to show more days using alcohol than expected female users. The control variables were unable to distinguish structural zeros (i.e. expected non-users) from zeroes that occurred by chance (i.e. expected users that didn’t consume alcohol in the past 30 days). In Model 2, gender was still associated with drinking days while distraction bias to alcohol was positively associated with a higher number of drinking days within the last 30 days (*p* = 0.004). Higher distraction bias scores tended to relate to decreased likelihood of being a non-user but did not reach statistical significance. In Model 3, gender (being male), age, distraction bias and craving scores were all significantly positively associated with increased number of drinking days. In the third and final model, age was the only variable significantly able to discern users from non-users (*p* = 0.026), showing that as age increases the likelihood of being a non-user decreases. Results of the regression analyses are presented in Table 2.

## Discussion

Our study shows that biased distraction towards alcohol-related cues among youth is associated with number of preceding drinking days. Participants with increased proclivity for distraction in the presence of alcohol cues, as indicated by slowed responses, tended to have increased alcohol use, but not necessarily higher degree of problematic use (AUDIT score). We found no main effect of stimulus type on response latency on Go trials or false alarms on NoGo trials, nor a statistically significant relationship between biased false alarms and drinking days or problematic alcohol use. Exploratory analyses pointed to evaluative craving scores as contributing a substantial and significant amount of explained variance in number of days drinking. The results reported here support the idea that both biased distraction towards alcohol cues and alcohol craving are associated with preceding drinking days, even when controlling for the effects of demographic variables (age, gender, and years of education). One of the primary strengths of the present study is the use of ZINB regression to separately model users and non-users as well as levels of consumption among users. Here, the results show that the variance in participants’ gender, age, their distraction to contextual alcohol cues, and their alcohol craving are significantly associated with the variance in number of drinking days within expected users, but that the variance in age is the only variable able to statistically discern whether a person is a user or non-user. This is also of theoretical interest for substance use research, as it suggests that other factors than distraction to alcohol cues are more pertinent to the status of being a user or non-user.

Our main finding is that the inclusion of distraction bias accounts for 11% additional variance, beyond what can be attributed to demographic variables, which alone accounts for 17%, and where only gender was a significant variable among these. Since both alcohol and neutral cues were task-irrelevant, slowed responding on alcohol cues compared to neutral can be interpreted as a distraction from the task-relevant (“P” versus “F”) discrimination. The demonstrated association contributes to the literature linking individual cognitive biases and individual differences in substance use [1, 4, 53] by showing a robust correlation between distraction bias to alcohol cues and number of days drinking in a sample of light and moderate-to-heavy young drinkers. The results can be interpreted in the framework of Robinson and Berridge’s (2001) model of drug addiction, suggesting that the alcohol cues had acquired a higher degree of emotional or incentive value for heavier drinking individuals, as a consequence of the rewarding effects of alcohol exposure [27, 28]. Our findings dovetail with recent studies reporting slower Go responses when emotional stimuli are used as distractors, which is commonly interpreted as emotional distractors drawing attention away from the cognitive task [26]. The abovementioned studies show stimulus conditions to have main effects on dependent measures, but in contrast we fail in our study to find similar effects using alcohol cues. We surmise, however, that the degree to which alcohol cues have acquired emotional valence or incentive value depend on the relative frequency of drinking in the preceding month. We also note that we find a strong correlation between distraction bias and craving, consistent with the notion that automatic cognitive biases and evaluative craving have a mutually occurring relationship [54].

Distraction bias, as it is measured in this modified Go/NoGo task, can be understood as conceptually overlapping with attentional bias as measured with a dot-probe task [4]. Should they overlap empirically regarding alcohol use, further studies might expect distraction bias to be impacted by chronic exposure to alcohol, as it is seen in AUD [8, 15] or acute exposure to alcohol [55, 56]. Future studies employing both tasks are warranted to investigate a possible correlation between these two measures or to disentangle their potential difference.

The findings are also consistent with cognitive theories of addiction, which assert that the drinker’s attention is involuntarily captured by alcohol-related cues and that this distractibility plays an important role in triggering the cognitive-behavioural processes leading to alcohol consumption. In this respect, attentional-distraction bias may serve as a novel neurocognitive biomarker that could possibly predict future alcohol use. Here, prospective studies would ideally be able to further clarify whether distraction bias can be used to predict non-problematic and problematic alcohol use, and the transition between them. It should however be noted that we did not find any correlation between distraction bias and problematic use as indicated by AUDIT.

We found no association between drinking days and inhibition bias measured as increased amount of failures to inhibit responding on trials with alcohol cues. The lack of association is consistent with a study employing the flanker task, showing increased latencies in the presence of alcohol cues, yet no effect of these cues on accuracy [57]. Similarly in a group of healthy young adults, the severity of binge drinking was not associated with inhibition on the CANTAB stop signal task [58]. One could also speculate that the degree of alcohol use correlates with specific variance in neural activity patterns as measured by functional magnetic resonance imaging (fMRI) during failed and successful response inhibition in the task without resulting in discernible overt behavioural measures, as has been demonstrated in younger age groups of drinkers [59, 60], for review see [61].

We show that, using these task parameters and this sample, a correlation between drinking days and alcohol specific lack of inhibition remains to be seen. In this regard, our results inform on recent proposals to compare bias modification effects of different variants of Go/NoGo training paradigms, using either inhibition in the context of alcohol or general inhibition training to decrease heavy drinking days [62]. Our study also highlights a lack of correlation between drinking days and specific inhibition to contextual alcohol cues.

The results of the explorative third regression model indicated that evaluative craving scores are associated with days drinking in the preceding month, beyond distraction bias and demographic variables. Studies on the relationship between alcohol craving and alcohol use have varied findings [3, 13, 29, 30]. We stress that our craving scores are derived from a novel approach, which is different from previous studies. The noted discrepancy in the literature might relate to interindividual differences in how subjective craving may relate to drinking behaviours, to which our method of calculating craving scores is potentially more sensitive. However, unlike prior studies [3], we did not simultaneously query participant’s pleasantness rating for neutral images as a way of controlling the craving scores. Thus, a limitation with our craving scores is that we cannot rule out that the relationship between craving scores and drinking days relates to a general tendency to rate images highly, although this is unlikely. One limitation with the design is that the neutral stimuli was not matched with the alcohol cues on emotional valence, and so it cannot be ruled out that the task measures general distraction to emotionally-valenced cues as opposed to specific alcohol cues. However, as a methodological strength in this study we used the craving score to individualize the stimuli for each participant, thus ensuring that participants were tested on alcohol stimuli that were salient to them.

Before concluding, the most notable limitation with the present study is that the cross-sectional nature of our design does not permit us to draw any causal inference between alcohol use and cognitive measures. Such relationships are ideally investigated in a longitudinal design, able to discern the relative predictive value of implicit and explicit cognitive biases to alcohol. In our case, despite the relatively large amount of variance explained by craving scores compared to distraction bias, it is not certain that interindividual variance in drinking behaviours across time can be explained to the same degree. Moreover, as the present study included young individuals without an AUD diagnosis, the generalization of the results to AUDs is limited. Further studies that directly assess distraction bias, craving and its relationship to traditional measures in a clinical sample is warranted to further clarify the applicability of the present findings.

## Conclusion

In conclusion, we show a novel measure of distraction bias specifically to alcohol cues and its robust association with number of drinking days within adolescents and young adults. The present study did not find evidence for a relationship between response inhibition failures to alcohol cues and drinking days. The results suggest that a beneficial avenue of research is the further investigation of distraction to alcohol cues and alcohol use behaviour across time and different AUD diagnostic populations. Identifying cognitive measures such as distraction bias and its relationship to alcohol use as it potentially leads to problematic use is crucial for the development of risk assessment and effective prevention strategies for alcohol addiction.

## Abbreviations

AIC: Akaike information criterion
AUD: Alcohol Use Disorder
AUDIT: Alcohol Use Disorder Identification Test
fMRI: Functional magnetic resonance imaging
GLM: Generalized linear model
LR: Likelihood Ratio
OLS: Ordinary least squares
RT: Reaction Time
SD: Standard deviations
ZINB: Zero-Inflated Negative Binomial
